# The first EGF domain of coagulation factor IX attenuates cell adhesion and induces apoptosis

**DOI:** 10.1042/BSR20160098

**Published:** 2016-06-03

**Authors:** Tomomi Ishikawa, Hisataka Kitano, Atsushi Mamiya, Shinichiro Kokubun, Chiaki Hidai

**Affiliations:** * Division of Physiology, Department of Biomedical Science, School of Medicine, Nihon University, 30-1, Oyaguchi Kami-cho, Itabashi-ku, Tokyo 173-8610, Japan; † Division of Dental Surgery, School of Medicine, Nihon University, 30-1, Oyaguchi Kami-cho, Itabashi-ku, Tokyo 173-8610, Japan

**Keywords:** adhesion, anoikis, apoptosis, epidermal growth factor, factor IX

## Abstract

Coagulation factor IX (FIX) is an essential plasma protein for blood coagulation. The first epidermal growth factor (EGF) motif of FIX (EGF-F9) has been reported to attenuate cell adhesion to the extracellular matrix (ECM). The purpose of the present study was to determine the effects of this motif on cell adhesion and apoptosis. Treatment with a recombinant EGF-F9 attenuated cell adhesion to the ECM within 10 min. De-adhesion assays with native FIX recombinant FIX deletion mutant proteins suggested that the de-adhesion activity of EGF-F9 requires the same process of FIX activation as that which occurs for coagulation activity. The recombinant EGF-F9 increased lactate dehydrogenase (LDH) activity release into the medium and increased the number of cells stained with annexin V and activated caspase-3, by 8.8- and 2.7-fold respectively, indicating that EGF-F9 induced apoptosis. Activated caspase-3 increased very rapidly after only 5 min of administration of recombinant EGF-F9. Treatment with EGF-F9 increased the level of phosphorylated p38 mitogen-activated protein kinase (MAPK), but not that of phosphorylated MAPK 44/42 or c-Jun N-terminal kinase (JNK). Inhibitors of caspase-3 suppressed the release of LDH. Caspase-3 inhibitors also suppressed the attenuation of cell adhesion and phosphorylation of p38 MAPK by EGF-F9. Our data indicated that EGF-F9 activated signals for apoptosis and induced de-adhesion in a caspase-3 dependent manner.

## Abstract (precis)

Activated coagulation factor IX (FIX) attenuated cell adhesion to the extracellular matrix (ECM) and induced apoptosis. This activity was localized to the first epidermal growth factor (EGF) domain, EGF-F9. Experiments with caspase-3 inhibitors revealed that attenuation of adhesion and apoptosis by EGF-F9 were dependent on caspase-3.

## INTRODUCTION

Anoikis is a type of programmed cell death that is induced by cell de-adhesion from the extracellular matrix (ECM) [1,2]. When cells lose integrin-mediated adhesion to the matrix, multiple pathways for apoptosis are activated [3,4]. Well-studied pathways of apoptosis are the so-called ‘extrinsic’ and ‘intrinsic’ pathways. Bcl-2 homology domain 3 (BH3)-only proteins play important roles in the intrinsic pathway for anoikis [5]. In this pathway, Bid and Bim are activated by cell detachment from the matrix and promote the assembly of Bax–Bak oligomers within the outer mitochondrial membrane (OMM). These Bax–Bak oligomers create a channel within the OMM, causing mitochondrial permeabilization and cytochrome *c* release. The release of cytochrome *c* leads to the formation of apoptosomes, which subsequently lead to activation of the anoikis effector caspase-3. In the extrinsic pathway of anoikis, loss of anchorage to the ECM leads to an increase in Fas and Fas-L expression [6,7]. Changes in cell shape during detachment can also induce anoikis through the extrinsic pathway, through membrane re-localization and activation of Fas [8,9]. Ultimately, both intrinsic and extrinsic pathways rely on activation of the effector caspase-3, which leads to activation of a proteolytic cascade for apoptosis [10].

Increasing attention has been paid to anoikis over the past 20 years. In particular, the regulation of cancer metastasis by anoikis has been a focus of study. Anoikis suppresses cancer cell survival in the blood stream and engraftment of cancer cells to the ECM in remote organs. The metastasis of many cancers, including prostatic cancer and breast cancer, has been reported to be suppressed by anoikis [11–14]. Because metastasis is a critical factor for prognosis of cancer patients, anoikis is a potential target of cancer therapy. In addition to cancer metastasis, anoikis has also been found to be involved in tissue homoeostasis, which ensures an internal steady state within tissues of an organism [15,16]. Anoikis plays an essential role in the formation of normal tubular structures in mammary glands, by deletion of cells that detach from the basement membrane.

Numerous studies have been performed to investigate the roles, mechanisms and applications of anoikis. Most of these studies have employed genetically altered animals and cells and/or blockers of adhesion molecules that enforce detachment of the cells from the ECM. Certainly, enforced detachment from the ECM induces the apoptosis of many types of cells. However, the triggers or the conditions that compel cells to detach from the matrix *in vivo* are not well known, nor is it known at what point cell detachment is positioned in apoptotic pathways.

An epidermal growth factor (EGF) motif is a consensus sequence that is characterized by the arrangement of cysteine residues. This motif is shared by more than 600 extracellular proteins and is considered to be biologically important [17,18]. Various subgroups of EGF motifs are known and 25% of all EGF motifs belong to the calcium-binding family of EGF motifs. Most of these calcium-binding EGF motifs have the consensus sequence, CX(D/N)XXXX(F/Y)XCXC where the D/N residues undergo β-hydroxylation. Kitano et al. [19] have proposed a novel subfamily that is included in this family. This subfamily shares the consensus sequence, CXDXXXXYXCXC. EGF motifs that are present in some membrane proteins such as Notch (AF508809), Jagged (AF171092) and delta-like 4 (DLL4) (AF253469), in some ECM proteins such as fibrillin (L29454) and Del1 (AF031524) and in some plasma proteins such as coagulation factor VII (FVII) (U44795), coagulation factor IX (FIX) (AK149372) and coagulation factor X (FX) (AF087644), belong to this subfamily.

The third EGF motif of Del1, an embryonic endothelial ECM protein, has the amino acid sequence CVDLGNSYLCRC, and the first EGF motif (EGF-F9) of the FIX, has the amino acid sequence CKDDISSYECWC. These two sequences have a positive effect on gene transfer efficiency via endocytosis [19]. Because replacement of the aspartic acid residue with an asparagine residue or replacement of the tyrosine residue with a phenylalanine residue abolishes this effect, the sequence CXDXXXXYXCXC could be a consensus sequence that defines a subfamily of EGF motifs. In addition to endocytosis enhancing activity, the third EGF motif of Del1 has been reported to induce apoptosis *in vitro* and *in vivo* [20]*.* This third EGF motif of De11 is adjacent to the second EGF motif, which has an RGD sequence that is an integrin binding domain [21,22]. The arrangements of these domains imply that apoptosis is induced by the consensus amino acid sequence, CXDXXXXYXCXC.

In the present study, the roles of cell adhesion in apoptosis induced by EGF-F9 were investigated. FIX consists of a light chain, an activation peptide and a heavy chain (trypsin domain) [23]. In the process of coagulation, cleavage of the activation peptide activates FIX. A heterodimer of the light chain and the heavy chain cleaves FX to activate it, thereby promoting coagulation. The light chain of FIX consists of a gamma-carboxyglutamate (Gla) domain, the first EGF (EGF-F9) and the second EGF motif. EGF-F9 has been found to be essential for FIX protease activity and to stabilize the Gla domain [24,25]. However, the effects of EGF-F9 on cells are not well known. Recently, Kitano et al. [26] reported that activated FIX (FIXa) attenuates cell adhesion to the ECM and that this activity is localized to EGF-F9. The present study investigated the signalling pathway of EGF-F9 and shows that EGF-F9 induces apoptosis *in vitro*. Finally, the position of cell de-adhesion in the apoptotic pathway induced by EGF-F9 is discussed.

## MATERIALS AND METHODS

### Cell line and culture

The human oral squamous cell carcinoma cell line A431 (A.T.C.C.) was grown in serum-free 64 medium [60% Opti-minimal essential medium (MEM) (Invitrogen), 40% LHC-8 medium (Invitrogen)]. Cells were cultured in 5% CO_2_ at 37°C.

### Reagents

**Figure 1:**
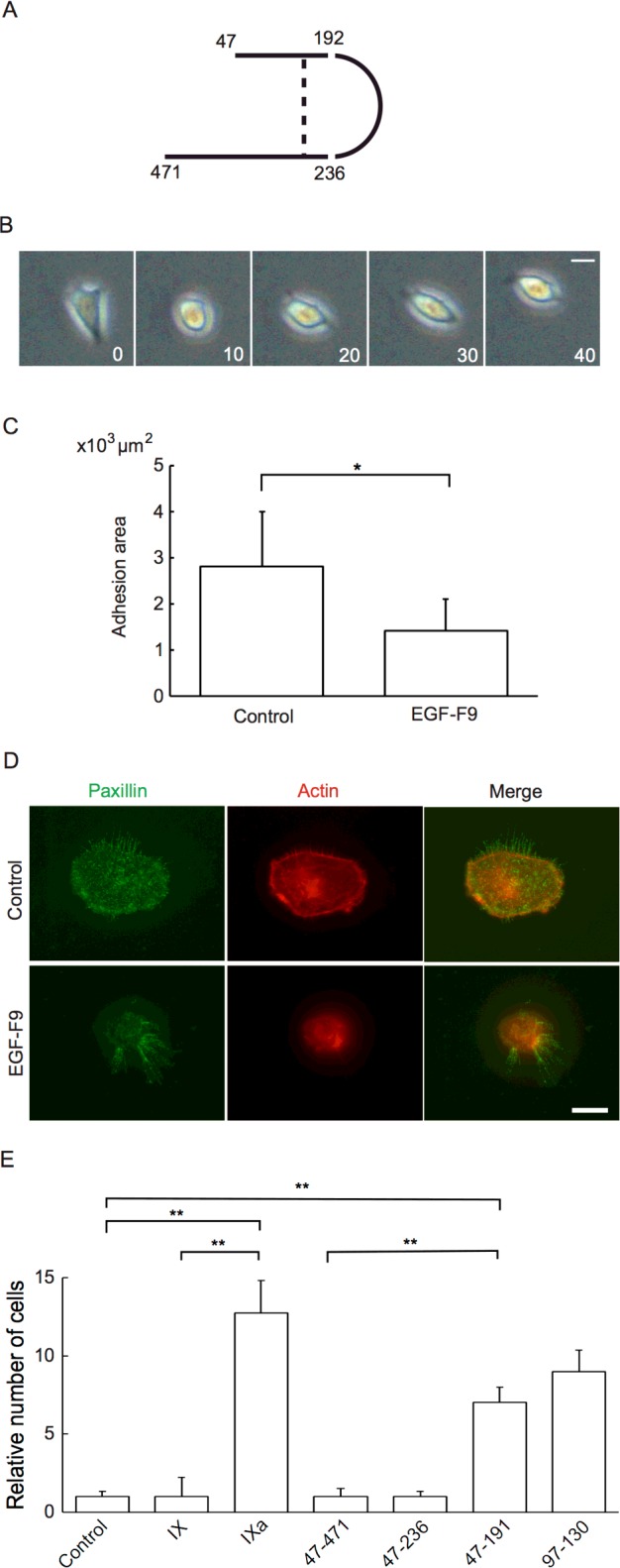
The effects of EGF-F9 on cell adhesion (**A**) Structure of F9. Amino acids 192 and 236 are cleavage sites for F9 activation. Numbers indicate amino acid residues. The dotted line indicates a disulfide bond. (**B**) A typical time-lapse recording of an A431 cell treated with 100 pmol/ml of AP-tagged EGF-F9. The numbers indicate time (min). The scale bar indicates 20 μm. (**C**) The adhesion area of A431 cells treated with 100 pmol/ml of the AP-tag (control) or the AP-tagged EGF-F9 for 10 min was quantified; *n*=15. (**D**) Typical immunofluorescence analysis of paxillin and actin in A431 cells treated with 100 pmol/ml of the AP-tag (control) or the AP-tagged EGF-F9 for 10 min. The scale bar indicates 10 μm. (**E**) De-adhesion assay in which attached A431 cells were treated with 100 pmol/ml of native inactive FIX or FIXa respectively or with 100 pmol/ml of the indicated AP-tagged recombinant mutant FIX deletion proteins. Following treatment, the dishes containing the cells were shaken and the number of detached cells was counted. The number of cells detached in the control dish was assigned a value of one. Numbers indicate amino acid residues. Values are indicated as means ± S.D.; *P<0.05, **P<0.01, *n*=6.

Recombinant alkaline phosphatase (AP)-containing fusion proteins were prepared as previously described [19,27–29]. Briefly, several F9 deletion mutants were generated using reverse transcription polymerase chain reaction (RT-PCR) and were cloned into the AP-tag4 vector (GenHunter) for production of AP-tagged FIX as a secreted protein in Chinese hamster ovary (CHO) cells cultured in 64 medium. The DNA constructs included mouse full-length FIX (amino acids 47–471, accession number P16294) without the N-terminal propeptide, a fragment of FIX lacking the heavy chain (amino acids 47–236), the FIX light chain (amino acids 47–191) and the first EGF domain of FIX (amino acids 97–130) (Figure 1A). An AP tag without FIX was used as a control. The AP activity in the conditioned medium was measured by adding 20 μl of the conditioned medium to a well in a 96-well plate. The enzyme reaction was initiated by adding 200 μl of substrate [1 mg/ml *p*-nitrophenyl phosphate (Sigma–Aldrich) in 1 mM MgCl_2_ and 1 M diethanolamine, pH 9.8] to each well, and the absorbance at 405 nm was measured after 30 min. Native human FIX and active FIX were purchased from Thermo Scientific. Antibodies that recognize caspase, cleaved caspase-3, actin, p38 mitogen-activated protein kinase (MAPK), phosphorylated p38 MAPK, phosphorylated MAPK 44/42 and phosphorylated c-Jun N-terminal kinase (JNK) were purchased from CST. An antibody against paxillin was purchased from Abcam. Alexa Fluor 488- or Alexa Fluor 568-labelled goat anti-rabbit antibodies and Alexa Fluor 568-labelled phalloidin were purchased from Invitrogen. Caspase-3 inhibitors V [Z-D(OMe)QMD(OMe)-FMK] and VII were purchased from Merck Millipore. Caspase-3 inhibitors (100 pmol/ml) were added to the culture medium 30 min before treatment with FIX recombinant proteins.

### Measurement of the area of cell adhesion

A431 cells were plated on fibronectin-coated coverslips for 1 h, and were then stained with Di-I and treated with 100 pmol/ml AP or an AP-tagged FIX fragment for 10 min at 37°C. The cells were fixed in PBS with 4% paraformaldehyde. Cross-sectional images of cells were obtained every minute at 60 × final magnification using a confocal microscope (Leica). The areas of the cross-sectional images of the adhered parts of the cells were measured using the AquaCosmos software package (Hamamatsu Photonics).

### Immunofluorescence

To investigate cell adhesion to the matrix, A431 cells were plated on fibronectin-coated coverslips for 1 h and were then treated with 100 pmol/ml AP or an AP-tagged FIX fragment for 10 min. at 37°C. The cells were fixed in PBS with 4% paraformaldehyde and were then permeabilized in PBS with 0.1% Triton X-100. The cells were then incubated with primary antibodies, and staining was detected with Alexa Fluor 488 or Alexa Fluor 568 conjugated secondary antibodies (Invitrogen). Alexa Fluor 568-labelled phalloidin was used to visualize polymerized actin. An Axioscope 2 (Carl Zeiss) equipped with an AxioCam (Carl Zeiss) was used to observe tissues and take photographs.

### De-adhesion assay

A431 cells were plated at a sub-confluent concentration in a 96-well plate and were cultured for 1 h. Recombinant proteins with AP tags (100 pmol/ml) were then added and the plate was shaken for 30 min at 1200 rpm on an OPM-103 microplate mixer (As One). Detached cells were aspirated using a pipette and were counted using a haemocytometer. Recombinant AP was used as a negative control.

Data were calculated relative to the number of detached cells in the negative control experiment, which was arbitrarily set to one. Results are expressed as the mean ± S.D.

### Detection of apoptosis

A lactate dehydrogenase (LDH) plus cytotoxicity detection kit (Roche Diagnostics) was used for semi-quantitative analysis of cell death. Released LDH in the medium was measured according to the manufacturer's protocol. The value obtained with the negative control sample treated with the control AP-tag protein was set to one. To stain apoptotic cells with annexin V, cells were incubated with Alexa Fluor 568 conjugated annexin V (Invitrogen) for 20 min at room temperature and were then fixed with 4% paraformaldehyde before microscopic examination. To count the number of cells, the polymerized actin in the cells was stained with Alexa Fluor 568-labelled phalloidin for 20 min at room temperature. The total number of cells and the number of cells stained with annexin V were measured with AxioVision (Carl Zeiss).

### Western blotting

A431 cells were cultured for 1 h in fibronectin-coated 24-well dishes, after which 100 pmol/ml AP- or an AP-tagged FIX fragment was added for 0, 5, 10 or 30 min at 37°C. The cells were then harvested in sample buffer. Proteins were separated by SDS/PAGE and were transferred to a polyvinylidene fluoride (PVDF) membrane (ATTO). Membranes were incubated with primary antibodies, followed by horseradish-peroxidase-conjugated secondary antibodies, and immunoreactive proteins were detected using a Western blotting ultra-sensitive HRP substrate (Takara). Chemiluminescence reactions were detected using Hyperfilm ECL (Amersham) or LumiCube (Liponix) and were evaluated with JustTLC software (Sweday).

### Statistical analysis

Wilcoxon's rank-sum test was used to assess the statistical significance of differences in means. The chi-square test was used to evaluate the equivalence of frequencies. Statistical significance was set at *P*<0.05.

## RESULTS

The effect of a recombinant AP-tagged EGF-F9 protein on cell adhesion to the ECM was analysed using A431 cells. Treatment of the cells with 100 pmol/ml EGF-F9 at 37°C induced rounding of the cells within 5–10 min (Figure 1B). This result suggested the attenuation of cell adhesion to the ECM by EGF-F9. However, gentle shaking of the dish did not induce detachment and floating of the cells. Therefore, to evaluate the effect of EGF-F9 on cell adhesion, the area of cell adhesion was examined using confocal microscopy. This analysis showed that the area of cell adhesion was decreased by 50% within 10 min of EGF-F9 treatment (Figure 1C). We next analysed cytoskeletal components of adhesion complexes after 10 min of EGF-F9 treatment by immunofluorescence analysis of paxillin and actin, using an anti-paxillin antibody and an Alexa Fluor-conjugated second antibody and Alexa Fluor 568-conjugated phalloidin respectively (Figure 1D). In control AP-tag treated cells, polymerized actin was localized at the periphery of the cells, in a circular pattern, at the cell-matrix adhesion surface and paxillin co-localized with actin. Following EGF-F9 treatment, the staining of actin and paxillin decreased within 10 min of treatment except for at the base of filopodia. A431 cells were found to predominantly express integrin α1β3 (results not shown). We therefore further analysed the effects of EGF-F9 on integrin α1β3 localization in these cells by double immunofluorescent staining of anti-integrin α1β3 with anti-paxillin or anti-vinculin antibody (Supplementary Figures S1A and S1B). In control cells, co-localization of integrin with paxillin or vinculin in a circular pattern at the cell periphery was observed. Treatment with EGF-F9 resulted in a decrease in their co-localization and fragmentation of the circular staining pattern. To investigate how AP-EGF-F9 mediates these effects in the context of the full-length protein, de-adhesion assays were performed using native FIX or deletion mutants of FIX (Figure 1D). De-adhesion activity was first compared between native FIX and FIXa. When the cells were incubated with native FIX or with a recombinant full-length FIX protein (100 pmol/ml) for 30 min, shaking of the dish did not detach the cells from the bottom of the dish. However, when the cells were incubated with FIXa, some cells did detach from the dish with shaking, indicating that FIXa attenuated cell adhesion to the matrix. Similar analysis of AP-tagged, recombinant FIX deletion mutant proteins indicated that deletion of the trypsin domain (amino acids 471–237) did not induce de-adhesion from the matrix but that deletion of the activation peptide (amino acids 192–236; constructs 47–191 and 97–131 in Figure 1E) did induce de-adhesion. These data suggested that the de-adhesion activity of EGF-F9 requires the same process of FIX activation as that which occurs for coagulation activity.

**Figure 2:**
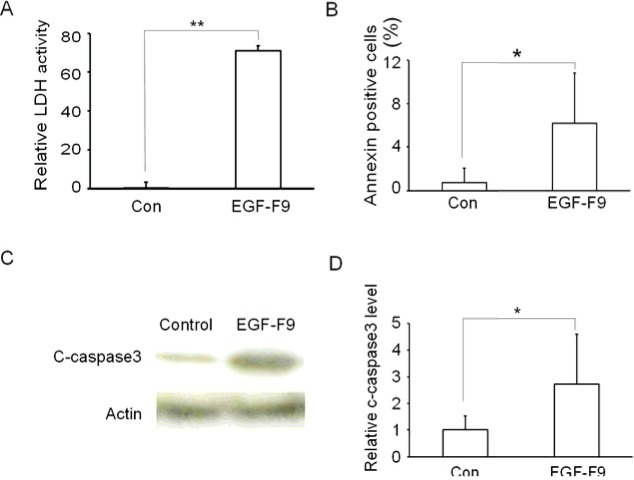
Induction of apoptosis by EGF-F9 A431 cells were treated with 100 pmol/ml of the AP-tag (control, Con) or of AP-tagged EGF-F9 for 10 min and apoptosis was assayed as follows: (**A**) Relative LDL activity in the medium was assayed; *n*=5. (**B**) The percentage of cells stained with annexin V conjugated with Alexa Fluor 488 was microscopically analysed; *n*=6. (**C**) Activation of caspase-3 was determined by Western blotting for cleaved caspase-3 (c-caspase-3). Actin was assayed as a loading control. (**D**) The relative amounts of cleaved caspase-3 were quantified. Values are indicated as means ± S.D.; *P<0.05, **P<0.01, *n*=3.

We next investigated if EGF-F9 might induce apoptosis, similar to the third EGF domain of Del1 that is homologous with EGF-F9. Treatment of A431 cells with recombinant EGF-F9 (100 pmol/ml) for 24 h induced LDH release into the medium, which is an indicator of cell death (Figure 2A). LDH release was significantly higher in EGF-F9 compared with control treated cells (*P*<0.01). Furthermore, the percentage of EGF-F9 treated cells that were stained with annexin V (another marker of apoptosis) was significantly higher than that of control cells (6.2% compared with 0.7% respectively; *P*<0.05) (Figure 2B). Western blotting also showed a 2.7-fold increase in the expression of cleaved caspase-3, which is an effector of apoptotic pathways, in EGF-F9 treated cells compared with control cells (Figures 2C and 2D). These results indicated that EGF-F9 induced apoptosis.

**Figure 3:**
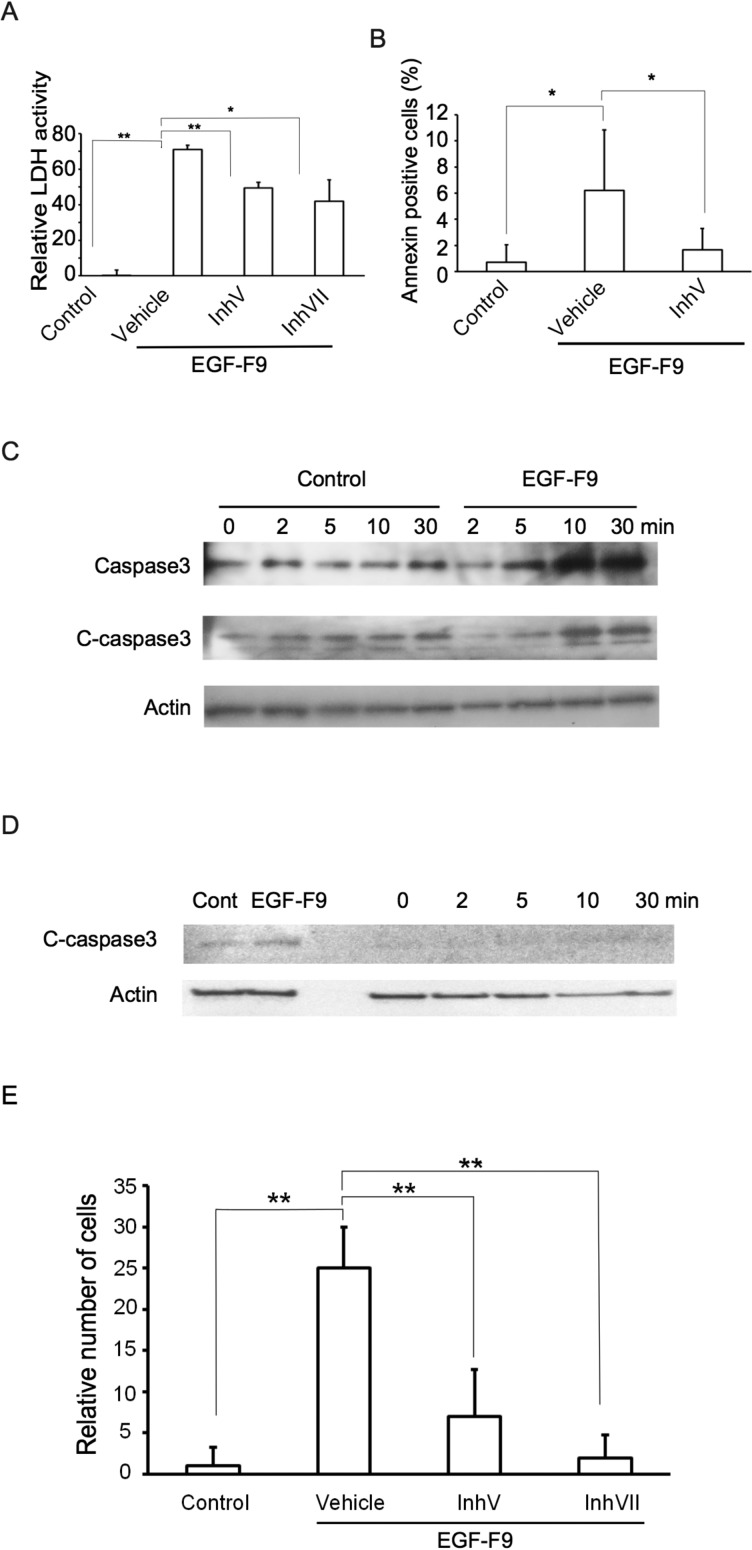
Caspase-3 dependent effects of EGF-F9 (**A**) The effect of caspase-3 inhibitors on EGF-F9-induced LDH activity in the medium. A431 cells were pretreated with DMSO (vehicle) or with 100 pmol/ml caspase inhibitor V (inhV) or VII (inhVII) for 30 min following which they were incubated with 100 pmol/ml of the AP-tag (control, Con) or of AP-tagged EGF-F9 for 10 min. LDH activity relative to the control was then assayed; *n*=3. (**B**) The effect of caspase-3 inhibitor V on the percentage of A431 cells stained with annexin V. The cells were treated as in (A), and analysed as in (Figure 2B); *n*=3. (**C**) Change in the cleavage of caspase-3 over time of EGF-F9 treatment (100 pmol/ml) of A431 cells was determined by Western blotting of full-length and cleaved caspase-3 (c-caspase-3). Actin was used as a loading control. (**D**) Western blotting analysis of the change in cleaved caspase-3 over time in cells prohibited from adhering to the bottom of the dish. (**E**) Effect of caspase-3 inhibitors on EGF-F9-induced de-adhesion. Cells were treated as in (A) except a de-adhesion assay was performed as described in Figure 1(E); *n*=5. Values are indicated as means ± S.D.; *P<0.05, **P<0.01.

Apoptosis induced by the third EGF domain of Del1 has been reported to be caspase dependent [20], and cleavage of caspase-3 was also observed with EGF-F9 treatment as shown in Figure 2(C). To confirm that apoptosis induced by EGF-F9 was caspase-3 dependent, the effect of treatment with a caspase-3 inhibitor, caspase-3 inhibitor V or VII on EGF-F9-induced apoptosis was assayed. Caspase-3 inhibitors (100 pmol/ml) were added to the medium and the cells were then cultured at 37°C for 30 min prior to EGF-F9 addition. Caspase-3 inhibitor V and VII significantly suppressed EGF-F9-induced LDH release into the medium, by 31% and 42% respectively (Figure 3A). Caspase-3 inhibitor V also significantly decreased the number of EGF-F9-induced annexin V stained cells by 57% (Figure 3B). Thus, apoptosis induced by EGF-F9 appears to be at least partially dependent on caspase-3.

As described above, EGF-F9 induced a de-adhesion effect after only 10 min incubation. We hypothesized that such de-adhesion could be a cause of apoptosis, the so-called anoikis. To determine if this was the case, we assayed the induction of caspase-3 cleavage by EGF-F9 over time using Western blotting. Unexpectedly, EGF-F9 induced caspase-3 cleavage within 5 min and within 10 min EGF-F9 had induced an increase in the level of uncleaved caspase-3 (Figure 3C). Based on previous reports of apoptosis induction, caspase-3 is activated from 30 min to a few hours after application of an apoptosis-inducing agent [30,31]. To examine if de-adhesion causes very early activation of caspase-3, the cells were cultured under conditions where they could not attach to a matrix, and cleavage of caspase-3 was assayed by Western blotting. For this purpose, A431 cells were harvested with trypsin/EDTA and then trypsin activity was neutralized with a trypsin inhibitor. The cells were then cultured in a glass-bottom dish to which A431 cells cannot adhere. Cell detachment with trypsin/EDTA decreased the amount of cleaved caspase-3 compared with attached cells (Figure 3D). Following detachment, there was no increase in cleaved caspase-3 over the following 30 min of incubation with EGF-F9. These data suggest that activation of caspase-3 isn't a result of de-adhesion. To test the hypothesis that caspase-3 activation is upstream of de-adhesion, a de-adhesion assay was conducted in the presence of caspase-3 inhibitors. Caspase-3 inhibitor V and VII suppressed EGF-F9-induced cell de-adhesion by 72% and 92% respectively (Figure 3E), indicating that caspase-3 activation may be upstream of EGF-F9-induced cell de-adhesion.

**Figure 4:**
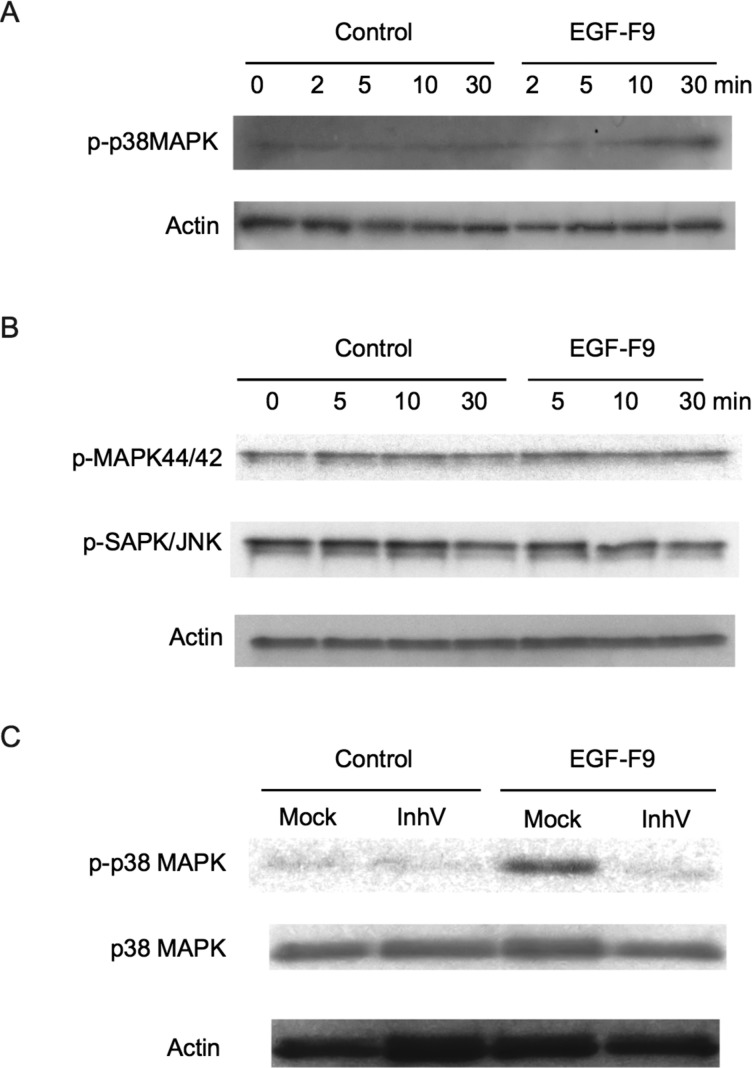
Effect of EGF-F9 treatment on the phosphorylation of MAPKs A431 cells were treated with 100 pmol/ml of the AP-tag (control) or of AP-tagged EGF-F9 for the indicated times following which phosphorylated p38MAPK (**A**) and phosphorylated MAPK 44/42 and JNK (**B**) were assayed by Western blotting. Actin was used as a loading control. (**C**) A431 cells were pretreated with or without (mock) 100 pmol/ml caspase-3 inhibitor V (inhV) for 30 min, and then treated with 100 pmol/ml of the AP-tag (control) or of AP-tagged EGF-F9 for 30 min. The level of phosphorylated (p-p38 MAPK) and total p38 MAPK was assayed using Western blotting.

Caspase-3 has been considered to be an effector caspase that is positioned downstream in the pathway of apoptosis induction. However, the above data indicated that caspase-3 was activated very quickly by EGF-F9 and that caspase cleavage was upstream of EGF-F9-induced de-adhesion. These results raised the intriguing possibility that caspase-3 activation by EGF-F9 might involve a rapidly activated apoptotic signalling pathway. We therefore investigated if this pathway might involve activation of the p38 MAPK. Western blotting indicated that treatment of A431 cells with EGF-F9 over a period of 30 min increased the level of phosphorylated p38 MAPK, but not that of phosphorylated MAPK 44/42 or JNK (Figures 4A and 4B). Treatment with the caspase-3 inhibitor V suppressed EGF-F9 induction of the phosphorylation of p38 MAPK by 60% (Figure 4C). These data suggest that the phosphorylation of p38 MAPK was positioned downstream of caspase-3 activation by EGF-F9.

## DISCUSSION

In the present study, we showed that FIXa attenuated cell adhesion to the ECM and that this activity was localized to the first EGF domain (EGF-F9). The present study therefore demonstrated that EGF-F9 has a biological function. Native FIX or recombinant FIX fragments containing the activation peptide did not display this activity, indicating that the activation of EGF-F9 in full-length FIX requires cleavage of the activation peptide, as occurs in the activation process for coagulation catalytic activity. In addition, EGF-F9 induced the apoptosis of A431 cells. This result was not unexpected because a homologous peptide, the third EGF domain of Del1, has apoptosis inducing activity.

EGF-F9-induced apoptosis was found to be caspase-3 dependent. The activation of caspase-3 was detected within 5 min after EGF-F9 treatment. There have been few reports of activation of caspase-3 at such an early time point after stimulation with an apoptosis inducing agent. Borutaite et al. [32] reported caspase-3 activation at an early time point (30 min) after stopping the circulation in ischaemic heart using a Langendorff-perfusion system. Because this increase was suppressed by an inhibitor of mitochondrial permeability transition (MPT), this activation of caspase-3 was considered to be dependent on cytochrome *c* release from the mitochondria. It has been reported that skin injury induces caspase-3 activation in gut epithelium within 30 min in *Drosophila* [33]. Because inhibition of caspase-3 results in death in this system, the activation of caspase-3 is essential for recovery from injury. Considering that FIX is strongly related to ischaemia and wound healing, these reports of early activation of caspase-3 suggest the existence of a possible common caspase-3 pathway that is required as a response to acute emergencies.

In EGF-F9 signalling, de-adhesion was localized downstream of caspase-3 activation. Some molecules that are involved in cell adhesion and migration have been reported to be substrates of caspase-3, including vinculin, E-cadherin and β-catenin [34–40]. Therefore, the proteolytic activity of caspase-3 on these adhesion molecules could be a cause of the attenuation of cell adhesion by EGF-F9. Additionally, caspase-3 catalyses poly (ADP-ribose) polymerase, p21-activated kinase 2, gelsolin, DNA fragmentation factor, α-fodrin and DNA-dependent protein kinase in the canonical pathway for apoptosis. This broad spectrum of caspase-3 substrates could be important in the induction of apoptosis by EGF-F9.

In the present study, activation of caspase-3 was essential for EGF-F9 induction of phosphorylation of the p38 MAPK, which is another key signalling protein in apoptosis [16,41,42]. It has been previously reported that p38 MAPK pathway is activated downstream caspase-3 [43]. The p38 MAPK is also involved in the intrinsic pathway for anoikis, in which it activates Bax [30]. Furthermore, the p38 MAPK is activated by detachment of cells from the ECM and it increases susceptibility to anoikis [7]. Thus, in the EGF-F9 signalling pathway, activation of the p38 MAPK could be related with signalling pathways that are activated following other apoptotic stimuli.

Inhibitors of caspase-3 only partially suppressed EGF-F9-induced apoptosis. This result suggested that EGF-F9 may also activate a caspase-3 independent pathway. It has been reported that anoikis is mediated via a caspase-independent pathway [44,45]. In this pathway, upon cell detachment from the ECM, the mitochondrial protein, Bit1, moves to the cytoplasm and induces caspase-independent apoptosis. Further investigation is required to determine if this pathway is involved in EGF-F9 signalling.

The combined data suggest that apoptosis induced by EGF-F9 involves caspase-3, de-adhesion, the p38 MAPK and a caspase-3 independent pathway. In the presence of EGF-F9, caspase-3 functions both upstream and downstream of cell de-adhesion. These results suggest that many factors interact to create a signalling network or a positive feedback signalling loop for the induction of apoptosis. There have been reports suggesting a complicated interaction among a number of factors and pathways for induction of apoptosis. For example cell detachment from the ECM increases the expression of FasL and activates the extrinsic pathway of apoptosis [7]. Activation of the p38 MAPK has been observed in the intrinsic pathway of apoptosis [30]. Caspase-3 has been found to activate caspase-9, which was previously considered to be upstream of caspase-3 [46]. From this point of view, de-adhesion could be perceived not only as a trigger but also as an enhancer of apoptosis.

The physiological roles of caspase-3 activation and apoptosis induction by FIX need to be addressed but include a number of possibilities. Firstly, apoptosis in injured tissue may be required for debridement [47]. Thus, physical induction of tissue wounding could distort tissue and such deformed tissue would need to be substituted by normal tissue. FIXa may help in the removal of damaged tissue with endocytosis. Secondly, attenuation of cell adhesion by EGF-F9 could enable cells to migrate into a wound. Wound healing requires the migration of many kinds of cells, including epithelial cells, fibrocytes and macrophages. It has been reported that the number of migrating macrophages is reduced in a wound induced by punch biopsy in F9 deficient mice [48,49]. Thus, EGF-F9 could be essential for normal cell migration into a wound. Thirdly, FIX may play some roles in the differentiation of cells and in the regeneration of tissues that are required for wound healing. In lower animals, such as *Hydra* and *Xenopus*, apoptosis increases during the process of regeneration [50–52]. Such apoptosis is rapidly detected by Terminal deoxynucleotidyl transferase dUTP nick end labeling (TUNEL) analysis within 1 h after injury. MAPK signalling in apoptotic cells increases secretion of the Wnt-ligand that activates compensatory proliferation of surrounding cells. Compensatory proliferation is effector caspase dependent in the differentiating eye tissue of *Drosophila* [53]. In mammals, caspase-3 has been reported to be essential for the differentiation of some cell lineages, including skin epithelial cells and haematopoietic cells. Okuyama et al. [54] has reported that a Notch-caspase-3 axis regulates the differentiation of keratinocytes. Activation of the p38 MAPK downstream of caspase-3 activation is essential for haematopoietic cell differentiation [43]. It has been reported that wound healing and regeneration in skin and liver is decreased in caspase-3 or caspase-7 deficient mice [55]. Thus, the apoptosis inducing activity of EGF-F9 could be involved in healing and regeneration at a site of injury. In addition to physiological responses in wound healing, de-adhesion and apoptosis induced by EGF-F9 could be a cause of vascular disease [56]. Thus, active FIX on endothelium could induce the detachment of endothelial cells and/or apoptosis in atheromatous lesions, leading to acute coronary disease.

In summary, in the present study, it was shown that EGF-F9 induces apoptosis, as does the third EGF domain of Del1 to which EGF-F9 is homologous. The amino acid sequence, CXDXXXXYXCXC, is probably a consensus sequence that would make this a subfamily of EGF motifs. This sequence is shared by FVII and FIX and by Notch related proteins. For further understanding of the functions of these proteins, the molecular mechanisms of this EGF motif should be investigated.

## Abbreviations

AP: alkaline phosphatase
BH3: Bcl-2 homology domain 3
CHO: Chinese hamster ovary
DLL4: delta-like 4
ECM: extracellular matrix
EGF: epidermal growth factor
FIXa: activated FIX
FVII: coagulation factor VII
FIX: coagulation factor IX
FX: coagulation factor X
Gla: gamma-carboxyglutamate
HRP: horseradish peroxidase
JNK: c-Jun N-terminal kinase
LDH: lactate dehydrogenase
MAPK: mitogen-activated protein kinase
MEM: minimal essential medium
OMM: outer mitochondrial membrane
RT-PCR: reverse transcription polymerase chain reaction
PVDF: polyvinylidene fluoride
TUNEL: Terminal deoxynucleotidyl transferase dUTP nick end labeling
